# White‐Light‐Emitting Melamine‐Formaldehyde Microspheres through Polymer‐Mediated Aggregation and Encapsulation of Graphene Quantum Dots

**DOI:** 10.1002/advs.201801432

**Published:** 2018-11-22

**Authors:** Youshen Wu, Hui Zhang, Aizhao Pan, Qi Wang, Yanfeng Zhang, Guijiang Zhou, Ling He

**Affiliations:** ^1^ Department of Chemistry School of Science Xi'an Jiaotong University Xi'an 710049 P. R. China; ^2^ Key Laboratory of Biomedical Information Engineering of Education Ministry Xi'an Jiaotong University Xi'an 710049 P. R. China

**Keywords:** graphene quantum dots, melamine‐formaldehyde, microspheres, white light emitting

## Abstract

Graphene quantum dot (GQD) encapsulated melamine‐formaldehyde (MF) polymer microspheres with uniform particle size and tunable high‐quality white‐light emissions are prepared via a polymer‐mediated GQD assembly and encapsulation strategy. In solution, GQDs are first aggregated with MF prepolymer through electrostatic interaction and further encapsulated inside the microspheres formed by polymerization of MF prepolymer under acid catalysis and heating. During this process, the aggregated GQDs are fixed in the MF polymer matrix with their emission extended from blue to full visible range, presenting bright white luminescence under ultraviolet excitation. The prepared white‐light‐emitting GQD‐MF microspheres exhibit uniform morphology with an average particle size of 2.0 ± 0.08 µm and their luminescence properties are effectively regulated by the doping concentration of GQDs in the MF polymer matrix. A series of white‐light‐emitting GQD‐MF microspheres with quantum yields from 0.83 to 0.43, Commission Internationale de L'Eclairage coordinates from (0.28, 0.28) to (0.33, 0.32), and color rendering index from 0.75 to 0.88 are obtained with excellent photostability and thermal stability. By dispersing the GQD‐MF microspheres in cross‐linked polydimethylsiloxane matrix, flexible film with dual functions of high‐quality white‐light‐emitting and light diffusion is obtained and successfully applied for white light‐emitting diode fabrication.

White‐light‐emitting materials have important applications in the fields of light‐emitting diode (LED) lighting, sensing, and other fields.^1^, ^2^, ^3^, ^4^, ^5^ Currently, practical white‐light‐emitting materials are mainly inorganic phosphors containing rare‐earth elements (REEs).^6^, ^7^, ^8^, ^9^ However, the mining and utilization of REE resources trigger many environmental and economic issues.^5^ Therefore, the development of low‐cost REE‐free luminescent materials with efficient, stable, and high‐quality white‐light emission is highly important. Various organic or carbon fluorescent materials including carbon quantum dots,^10^, ^11^, ^12^, ^13^ organic dyes,^14^, ^15^, ^16^, ^17^, ^18^ conjugate polymers,^19^, ^20^ and graphene quantum dots (GQDs)^21^, ^22^, ^23^, ^24^, ^25^ have been used to develop white‐light‐emitting materials. However, these materials still have many shortcomings in luminous efficiency, color, and stability.^2^, ^4^

GQDs are receiving extensive attention because of their superior properties, such as size‐tunable emission, high chemical, and optical stability, as well as good biocompatibility.^26^, ^27^, ^28^, ^29^, ^30^ Interestingly, the luminescence properties of GQDs can be regulated not only by their size and modifications but also by their assembly state.^26^, ^31^, ^32^ Aggregation of GQDs can result in redshift and broadening of their emission spectra.^25^, ^33^ Through controlled GQD aggregation in aqueous solution regulated by GQD concentration and solution pH and temperature, Ghosh and Prasad achieved white‐light emission using a single kind of unmodified GQDs.^25^ Compared with materials prepared from multiple fluorophores, the materials obtained by controlled aggregation of a single kind of GQDs have several significant advantages. First, their emission spectra can cover the entire visible light region and they have good color rendering. Second, their emitting color will not change during continuous irradiation, unlike materials containing multiple fluorophores because of the different fluorophores have different bleach rates. Third, the preparation procedure is facile and does not involve the fabrication of multilayer structures, which is suitable for large‐scale applications.

However, existing reports on the emission modulation of GQDs through their assembly were mostly implemented in aqueous solutions.^25^, ^33^ The aggregation state of GQDs, and therefore their emission properties, is sensitive to their concentration and the solution pH, which limits their practical application for LED lighting. If the GQDs are encapsulated inside the polymer matrix in a controlled manner and their luminescent properties regulated by their aggregation state, solid‐state white‐light‐emitting materials with high stabilities can be developed. The presence of a polymer matrix not only regulates the aggregation state of GQDs but also prevents the influence from other quenching factors, which can effectively improve the luminescence properties of the materials. The melamine‐formaldehyde (MF) resin is highly transparent in the UV–vis range, and is of high thermal stability.^34^, ^35^ The highly branched and cross‐linked internal structure also enables the stable encapsulation of various fluorophores,^35^ which is of interest for the development of various fluorescent micro/nanomaterials.^35^, ^36^, ^37^

In this study, we developed a polymer‐mediated GQD aggregation and encapsulation strategy using MF microspheres as a polymer matrix (**Scheme** **1**). GQDs were initially mixed with water‐soluble MF prepolymer in solution and then the GQD‐MF prepolymer complex obtained was further cross‐linked to form GQD‐encapsulated MF microspheres. During this process, the GQDs were aggregated and fixed in the MF polymer matrix, which caused their emission spectrum to extend from the blue region only to the full visible range, resulting in a bright white luminescence under UV excitation. The aggregation states of the GQDs, or equivalently their emission color, can be regulated by the GQD doping concentration in the MF matrix. GQD‐MF microspheres with a quantum yield (QY) of 43%, Commission Internationale de L'Eclairage (CIE) 1931 chromaticity coordinates of (0.33, 0.32), and a color rendering index (CRI) of 0.88 were successfully prepared with high GQD doping concentration of 4 wt%. The prepared GQD‐MF microspheres showed a uniform size distribution with an average particle size of 2.0 ± 0.08 µm and excellent photostability and thermal stability. By dispersing the GQD‐MF microspheres in a cross‐linked polydimethylsiloxane (PDMS) matrix, a flexible film with both a high‐quality white‐light emission and good light diffusion was prepared and applied to fabricate a remote planar white LED, which demonstrated the strong potential this material has for solid‐state lighting.

**Scheme 1:**
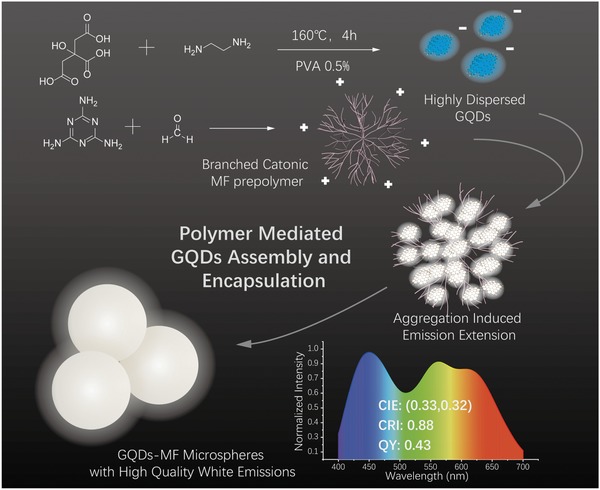
Preparation of GQD‐MF microspheres with high‐quality white emissions through polymer‐mediated GQD aggregation and encapsulation.

GQDs with a high QY were prepared through a bottom‐up method with minor modifications.^38^ GQDs with up to 55% QY and good dispersibility were synthesized from hydrothermal reaction of citric acid and ethylenediamine using polyvinyl alcohol (PVA) as a dispersant. PVA is an amphiphilic water‐soluble polymer, which could adsorb onto GQDs to improve their dispersibility in aqueous solutions,^39^ thereby preventing the agglomeration of GQDs during the separation and purification process, which is crucial for further analysis and regulation of GQD aggregation state. High‐resolution transmission electron microscope (HRTEM) and atomic force microscopy (AFM) were used to determine the morphology and structure characteristics of the prepared GQDs. As shown in **Figure** **1**A1, the GQDs were well dispersed with a Gaussian size distribution and an average size of 3.57 ± 0.31 nm (Figure 1A2). HRTEM images confirmed the crystalline nature of the prepared GQDs and revealed with a lattice space of 0.24 nm (Figure 1A3), which corresponds to the (1120) of graphite. Possible aggregation of the GQDs was investigated by AFM. Figure S1 (Supporting Information) shows that the topographic heights of the GQDs are mostly <0.7 nm, indicating that the prepared GQDs are generally single layered.^33^

**Figure 1:**
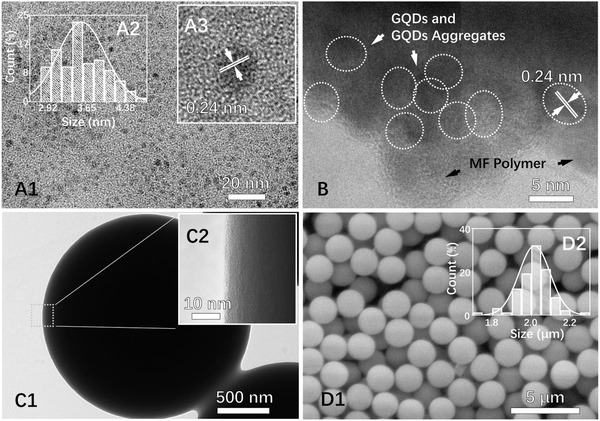
HRTEM images of A1,A2) GQDs and A3) the related size distribution. HRTEM images of C1,C2) GQD‐MF microspheres and B) pulverized GQD‐MF microsphere. SEM image of D1) GQD‐MF microspheres and D2) the related size distribution.

X‐ray photoelectron spectroscopy (XPS) was used to determine the GQD composition. As shown in Figure S2A (Supporting Information), C_1s_, N_1s_, and O_1s_ signals were observed at 284, 400, and 530 eV, respectively, indicating successful N‐doping. The high‐resolution C_1s_ XPS spectrum can be fitted into three Gaussians centered at 284.5, 286.1, and 288.6 eV, which correspond to the sp^2^ carbon (C—C/C=C) in graphene, the sp^3^ carbon in C—O and C—N, and the C=O in carboxyl group, respectively (Figure S2B, Supporting Information). The high‐resolution N_1s_ XPS peak of GQDs can be fitted with two Gaussians centered at 399.8 and 401.5 eV, which correspond to the pyrrolic N and graphite N (Figure S2C, Supporting Information). The UV–vis absorption and fluorescence spectra of the prepared GQDs in aqueous solution were acquired (Figure S3A, Supporting Information). Two clear absorption bands were observed at 230 and 350 nm, which are related to the π → π* and n → π* transitions of C=C and C=O bonds,^40^ respectively. The GQD solution demonstrated a blue emission with peak at 450 nm and full‐width at half maximum of 90 nm.

The GQDs obtained were dispersed in water and then mixed with water‐soluble MF prepolymer. Zeta potential of GQDs and prepolymer were measured under varied pH conditions (pH 2 to pH 12). As shown in Figure S4 (Supporting Information), zeta potential of GQDs decreases from −4.1 ± 3.3 mV to −21.4 ± 6.2 mV as pH increases from 2 to 12, while zeta potential of MF prepolymer decreases from 82.1 ± 15.2 mV to −11.2 ± 6.1 mV as pH increases from 2 to 12; these measurements are consistent with previously reported zeta potential analysis results of GQDs and melamine‐formaldehyde resin colloids.^34^, ^41^ In the pH range of 2 to 4 (which was used for MF microsphere preparation), the GQDs and MF prepolymer will aggregate with each other due to charge neutralization. In solution, the negatively charged GQDs were adsorbed and aggregated on the branched cationic MF prepolymer through dominant electrostatic interactions. As shown in Figure S3C (Supporting Information), absorption of the solution changes with the formation of the GQD‐MF prepolymer complex. The absorption peak blue shifted from 350 to 344 nm, and the absorption at 450 nm is also enhanced. These changes are very similar to the solvent dependence of the carbon quantum dot absorption spectra,^42^ which successfully confirmed the binding of GQDs and MF prepolymer.

Under acidic catalysis and heating, the GQD‐MF prepolymer complex was polymerized further and cross‐linked to prepare GQD‐encapsulated MF microspheres. The GQD‐MF microspheres formed were separated by centrifugation, and absorption analysis of the supernatant showed that more than 99% of the GQDs added were encapsulated in the microspheres, indicating strong affinity between the GQDs and the MF polymers. After washing and drying, the microspheres obtained turned into fine powders without any agglomeration. Under the excitation of 365 nm, the aqueous suspension and solid powder of the GQD‐MF microspheres presented a bright white fluorescence (**Figure** **2**A,B). To the best of our knowledge, this is the first time fluorescent microspheres were obtained using GQDs.

**Figure 2:**
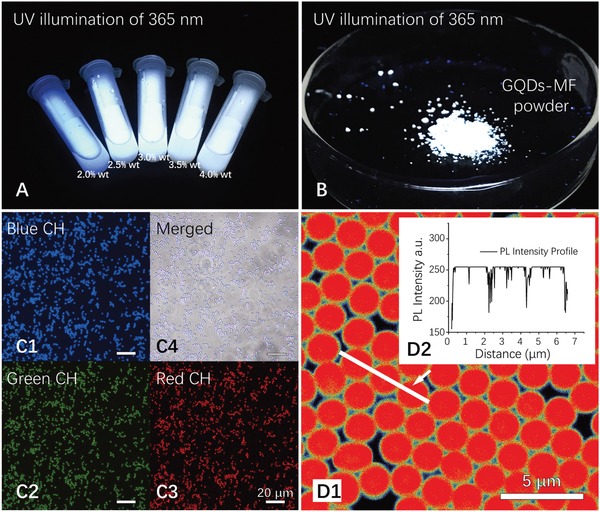
A,B) Aqueous suspension and powder of GQD‐MF microspheres under UV illumination at 356 nm. C1–C4) Fluorescence microscopy images of GQD‐MF microspheres, with a UV excitation of 360 ± 15 nm and a blue (460 ± 30 nm), green (520 ± 25 nm), or red (590 ± 35 nm) emission filter. SLSCM image of GQD‐MF microsphere with GQD doping concentration of 4.0 wt% D1,D2) PL intensity profile of three selected microspheres, excited with 405 nm laser, using emission collection range of 420–650 nm.

The morphology and composition of the GQD‐MF microspheres were characterized by HRTEM, scanning electron microscopy (SEM), fluorescence microscopy, super‐resolution laser scanning confocal microscope (SLSCM), UV–vis absorption, and Fourier‐transform infrared spectroscopy (FTIR). HRTEM was employed to characterize the composition and structure of the prepared GQD‐MF microspheres. As shown in Figure 1C1,C2, the GQD‐MF microspheres showed smooth spherical shape and have a densely cross‐linked internal structure. However, the microspheres are too thick for HRTEM characterization, and due to the similar atomic composition and electron density of GQDs and MF polymer, the distribution of GQDs in the MF polymer matrix cannot be directly observed from the HRTEM images. To overcome these problems, the GQD‐MF microspheres were pulverized into small pieces that can be easily penetrated by electron beams, and grids were used instead of the carbon support films for sample preparation. In the HRTEM images obtained, the GQDs and MF polymer matrix can be identified and distinguished by their different crystal states, and GQD aggregates were successfully observed (Figure 1B). This revealed the binding state of GQDs to the MF polymer and confirmed the aggregation of GQDs within the MF polymer.

Morphology and size distribution of the prepared series of GQD‐MF microsphere samples were analyzed with SEM. As shown in Figure 1D1, the GQD‐MF microspheres prepared with GQD doping concentration of 4.0 wt% have a uniform size distribution with an average particle size of 2.01 ± 0.08 µm. As shown in Figure S5 (Supporting Information), GQD‐MF microspheres prepared using GQD doping concentrations of 2.0, 2.5, 3.0, and 3.5 wt% had an average particle size of 2.00 ± 0.05, 2.02 ± 0.06, 2.01 ± 0.08, and 2.01 ± 0.06 µm, respectively. This indicated that GQD encapsulation had little effect on the particle size and morphology of the MF microspheres. The highly uniform particle size and morphology of the GQD‐MF microspheres prepared suggests a high potential for labeling, imaging, and luminescence.

As previously reported, the MF microspheres were formed through colloidal aggregation.^34^, ^35^ The fluorescent molecules added can be evenly doped inside the microspheres during this process because of the highly cross‐inked internal structure of the melamine resin.^35^, ^36^ Compared with dye molecules, GQDs have a larger average size of 3.57 ± 0.31 nm and can therefore be easily trapped into the cross‐linked polymer network of the MF microspheres. No leakage was observed during the repeated separation and washing treatments. Fluorescence microscopy and SLSCM were employed to investigate the microscopic emission characteristics of the GQD‐MF microspheres. As shown in Figure 2C,D, the GQD‐MF microspheres had a uniform and bright fluorescence under 365 nm UV and 405 laser excitation, which confirmed the uniform distribution of GQDs in the prepared microspheres. The fluorescence microscopy images also showed that the microspheres have a strong emission in the blue, green, and red channels (Figure 2C1–C3). In addition, the fluorescent images obtained using different channels are highly coincident (Figure 2C4), and fluorescence emission intensity from each microsphere is also highly consistent (Figure 2D2). This proved that the white emission of the obtained material is derived from the high uniformity of each individual microsphere.

As shown in Figure S3B (Supporting Information), the UV–vis absorption spectra of GQD‐MF microsphere samples showed no obvious peaks, and their absorptions are proportional to the GQD doping concentrations (B), which confirmed the quantitatively encapsulation of GQDs in the MF microspheres. The FTIR spectra of the GQDs, GQD‐MF microspheres, and blank MF microspheres were compared (Figure S6, Supporting Information). Since the mass fraction of GQDs in the MF microspheres is in the range of 2.0–4.0 wt%, the infrared absorption spectrum of the GQD‐MF microspheres was expectedly very close to that of the blank microspheres. This indicated that the encapsulation of GQDs had little influence on the composition of the polymer matrix.

As mentioned above, the prepared GQD‐MF microspheres showed bright white fluorescence, while the fluorescence of GQD solution is blue, which indicated that the emission characteristic of GQDs has changed during microsphere preparation. Fluorescence of GQDs is affected by various factors such as size, doping, edge groups, and aggregation.^23^, ^32^, ^42^, ^43^ During the preparation of GQD‐MF microspheres, the GQD emission could be affected by various factors including heating, solution pH change, aggregation, and chemical coupling. Therefore, to understand the white‐light‐emitting mechanism of the GQD‐MF microspheres, influences of heating, pH changes, and other factors on the fluorescence of GQDs were analyzed.

As shown in Figure S7A (Supporting Information), the fluorescence emission intensity of GQD solution decreases from 100% to 61% as temperature increases from 25 to 90 °C; however, this change is recoverable, and heating does not bring about a change in the shape of the emission spectrum of the GQDs. As shown in Figure S8 (Supporting Information), the fluorescence emission intensity of GQD solution and GQD suspension decreased as solution pH decreases from 10 to 2, with their emission spectra shape unchanged. These results indicated that the changes in temperature and solution pH during the preparation of microspheres are not the main cause of the white‐light‐emission of the GQD inside the microspheres. MF resin has good thermal stability and can withstand solvent soaking, but under the action of strong acidity, the MF microspheres will break down into soluble small molecules.^44^ The decomposed MF can be separated from solution by dialysis, and this process has been widely used in the preparation of microcapsules and hollow particles.^45^ Based on the decomposability of MF microspheres, an experiment was performed to analyze the possible fluorescence change mechanism of GQDs during the GQD‐MF microsphere preparation. The prepared GQD‐MF microspheres were immersed in strong acidity (1 m HCl) solution and ultrasonically pulverized to disintegrate the MF matrix and the decomposed MF polymer was removed by dialysis. The pH of the re‐acquired GQD solution was adjusted, and fluorescence characteristics of GQDs before and after this process were measured and analyzed.

As a result, the re‐extracted GQD solution showed unchanged emission peak of 450 nm, and slightly expanded full width at half maximum (FWHM) of 102 nm (Figure S9, Supporting Information). The normalized absorption and excitation spectra of the re‐extracted GQDs kept unchanged, either. These results indicated that the white‐light‐emission of GQDs inside GQD‐MF microspheres should not be attributed to the change of their chemical structure.

The changes in the GQD aggregation state during microsphere preparation could be observed by the changes in fluorescence emission of the GQDs (**Figure** **3**B1). Aggregation occurred when the GQDs were mixed with the MF prepolymer. The emission of individual GQDs at 450 nm decreased and new emission bands appeared between 500 and 700 nm. When the microspheres were formed, the fluorescence emission spectrum changed further, which indicated that the aggregation degree of the GQDs was further increased. As revealed by emission spectra, aggregation degree of the GQDs inside the MF microspheres, and therefore their emission, is related to the GQD doping concentration (Figure 3B2). As GQD doping concentration increased, the emission of individual GQDs at 450 nm decreased continuously, whereas the aggregation‐induced emission band at longer wavelengths increased.

**Figure 3:**
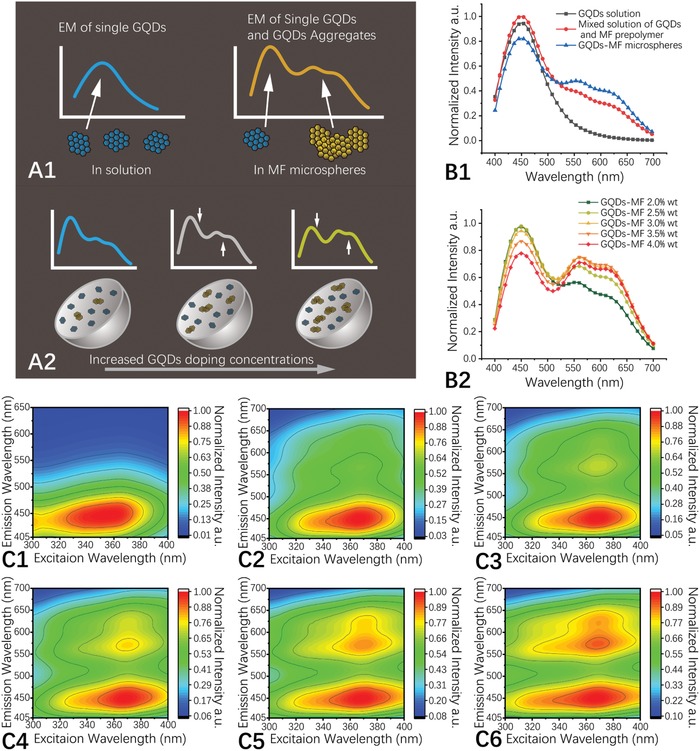
Schematic illustration of A1) GQD aggregation mediated white‐light‐emitting mechanism and A2) GQD doping concentration related emission color. A) Emission spectra of GQD solution (0.1 wt%), a mixed solution of GQDs and MF prepolymer, and of the GQD‐MF microspheres, excited at 360 nm. The spectra are normalized by the mass concentration of GQDs. B) Emission spectra of GQD‐MF microspheres with different GQD doping concentrations, excited at 360 nm. The spectra are normalized by the mass concentration of the microspheres. C1–C6) 3D fluorescence spectra of GQD solution (0.1 wt%) and MF‐GQD microspheres with GQD doping concentrations of 2.0 wt%, 2.5 wt%, 3.0 wt%, 3.5 wt%, and 4.0 wt%.

With these experimental and analysis results, we proposed a GQD aggregation regulated white‐light‐emission mechanism: When GQDs are mixed with MF prepolymer and incorporated inside the MF microspheres, partially aggregation of GQDs then makes the GQD‐MF microspheres exhibit white‐light emission (Figure 3A1). This model can also give a good explanation for the GQD doping concentration related emission color changes: With the increase of GQD doping concentration, the proportion of aggregated GQDs inside GQD‐MF microspheres also increased (Figure 3A2).

The encapsulation of then GQDs in the MF matrix also increased their luminescence efficiency. The QY of GQD‐MF prepared with a GQD doping concentration of 2.0 wt% increased to 151% compared to that of a 0.1 wt% GQD solution (Table S1, Supporting Information). This indicated that the encapsulation of the polymer matrix reduced the various quenching effects in the GQDs, which is very valuable for the preparation of highly efficient stable solid‐state luminescent materials using GQDs. As GQDs were mixed with the MF prepolymer, aggregation occurred, the emission of individual GQDs at 450 nm decreased, and new emission bands appeared at 500–700 nm. When the microspheres were formed, the fluorescence emission spectrum changed further, which indicated that the aggregation degree of GQDs was further increased.

The luminescent properties of the GQD‐MF microspheres were further analyzed with 3D florescence spectroscopy and time‐resolved fluorescence spectroscopy. To investigate the influence of excitation wavelength on the fluorescence of GQD‐MF microspheres, 3D fluorescence spectra of the prepared GQDs and MF‐GQD microspheres were measured. As shown in Figure 3, under UV excitation of 300–400 nm, emissions of GQD solution are mainly located in the blue region of 405–500 (Figure 3C1), while MF‐GQD microspheres showed expanded emissions in full visible range of 405–650 nm (Figure 3C2–C6). The maximum excitation wavelengths of the GQD solution and GQD‐MF microspheres are located at 358, 365, 368, 369, 369, and 367 nm, respectively, and their emission shapes show little excitation wavelength dependency in this excitation range. As shown in Figure S10 (Supporting Information), the average lifetimes of the GQD‐MF microspheres (8.1 ns for a GQD doping concentration of 2.0 wt%) were shorter than that of a similar GQD solution (10.2 ns, 0.1%) and continuously decreased with increasing GQD doping concentrations (from 8.1 to 5.5 ns as GQD doping concentration increased from 2.0 wt% to 4.0 wt%). This could also be attributed to increased GQD aggregation at higher GQD doping concentrations.

The spectral properties of the series of GQD‐MF microspheres as white‐light‐emitting materials for LED applications were analyzed further. The results showed that the luminescent colors of the prepared GQD‐MF microspheres were all located in the white area of the CIE 1931 chromaticity diagram under a UV excitation of 360 nm (**Figure** **4**A). The CIE 1931 chromaticity coordinates of the GQD‐MF microspheres prepared with GQD doping concentrations of 2.0%, 2.5%, 3.0%, 3.5%, and 4.0 wt% were arranged in sequence along the black body radiation curve and were (0.28, 0.28), (0.30, 0.29), (0.31, 0.32), (0.32, 0.32), and (0.33, 0.32), respectively. The corresponding correlated color temperatures (CCTs) were 10810, 7814, 6653, 5969, and 5638 K, respectively. Furthermore, a broadband emission would help to overcome major drawbacks of many lanthanide phosphors including a low CRI value. The CRI values of the series of GQD‐MF microspheres prepared were 0.75, 0.81, 0.84, 0.87, and 0.88 (Figure 4B1–B5). Compared with some recently developed GQD‐based white‐light‐emitting materials (Table S1, Supporting Information), the series of GQD‐MF microsphere materials has obvious advantages in quantum yield, CIE, and CRI, and is of significant practical value in the field of LED lighting. GQD‐MF microspheres prepared with a high GQD doping concentration of 4.0 wt% showed a high‐quality white emission. It had a QY value of 0.43, a CRI value of 0.88, a CCT value of 5638 K, and CIE 1931 chromaticity coordinates of (0.33, 0.32), which was very close to those of ideal white light (0.33, 0.33). Therefore, this doping concentration was selected to fabricate a white‐light‐emitting device.

**Figure 4:**
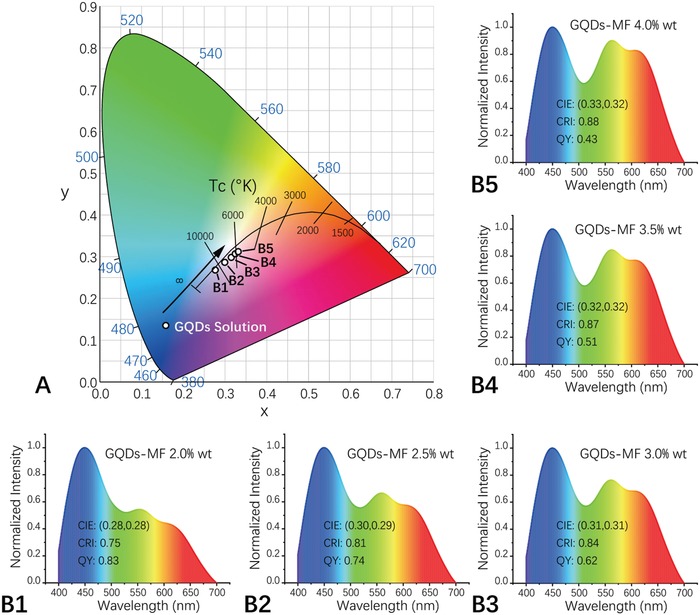
A) CIE 1931 chromaticity coordinates and B) corresponding emission spectra of the GQD‐MF microspheres prepared with different GQD doping concentrations, excited at 360 nm.

Stability has an important influence on the practical application of white‐light‐emitting materials. Compared with commercial inorganic white phosphors, the stability of many white‐light materials using organic fluorophores still has several shortcomings, including a low luminous efficiency at high temperatures and excessive photobleaching.^4^ The thermal stability of the GQD‐MF microspheres was investigated by temperature‐related fluorescence analysis. The results showed that the GQD‐MF microspheres have a good thermal stability, and their emission intensity and spectral characteristics do not change over a temperature range of 25–90 °C (Figure S7B, Supporting Information), whereas the emission of the GQD solution decreased to 62% when the temperature increased from 25 to 90 °C (Figure S7A, Supporting Information). In addition, fluorescence spectroscopy analysis also demonstrated that the luminescent properties of the GQD‐MF microspheres kept unchanged after drying for 24 h at 150 °C.

GQD have a higher photostability than fluorescent materials like organic dyes and CdTe QDs.^46^ The stability of the GQD‐MF microspheres prepared under continuous UV illumination was evaluated. The photobleaching results showed that the GQD‐MF microspheres and the GQD solutions were both stable. Their emission intensity and color kept unchanged during continuous UV irradiation for 3 h, whereas fluorescent dyes (Fluorescein and Rhodamine 110) were significantly bleached (Figure S11, Supporting Information). The dispersion and stability of GQD‐MF microspheres in various solvents have been determined as well. The GQD‐MF microspheres are stably dispersed with unaltered emission characteristics (**Figure** **5**A) and without dissolution or swelling in most common polar solvents, including water, tetrahydrofuran (THF), dimethylformamide (DMF), dimethyl sulfoxide (DMSO), dichloromethane, and chloroform. Such stability of the GQD‐MF microspheres further confirmed their potential practical advantage in LED fabrication.

**Figure 5:**
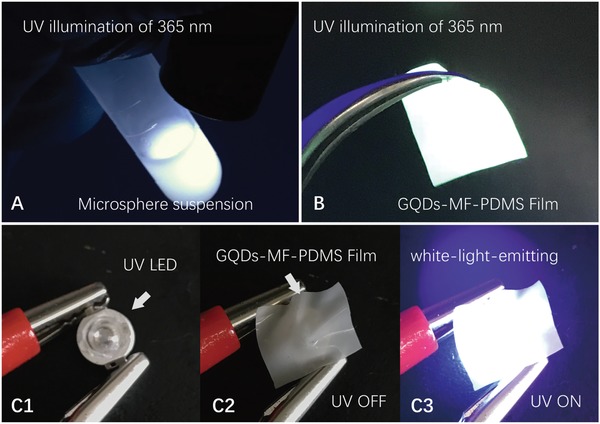
A) Dichloromethane dispersion of GQD‐MF microspheres. B) GQD‐MF microsphere embedded white‐light‐emitting PDMS flexible film. C1–C3) Remote planar white light‐emitting device prepared with a 365 nm UV LED chip and the GQD‐MF‐PDMS flexible white‐light‐emitting film.

Typically, single or multiple layers of luminescent materials are coated on UV or blue LED chips to construct white LED illuminators.^5^ To improve the efficiency of white LED devices, remote phosphor packaging technology has been developed.^47^ This technology helps the heat dissipation of the chip, and can improve the utilization efficiency of the excitation light, as well as improve the uniformity of the illumination.^48^, ^49^, ^50^ The good stability and dispersibility of GQD‐MF microspheres make it easy to mix with a variety of polymer materials (such as PVA, poly‐methyl‐methacrylate (PMMA), and PDMS) in organic solvents to prepare white‐light‐emitting blocks or film materials. The micrometer‐sized particle and high refractive index of the GQD‐MF microspheres also impart strong light scattering to the obtained white‐light‐emitting materials (Figure S12, Supporting Information).^51^, ^52^ By dispersing and embedding the GQD‐MF microspheres with commercial Dow Corning SYLGARD 184 silicone elastomer kit (microsphere: PDMS mass ratio of 5:100), a flexible white‐light‐emitting GQD‐MF‐PDMS film was prepared. The prepared film had a milky white appearance and good flexibility. It can be stretched and wrapped on the surface of a specific device. Given the light diffusion effect of the film, a uniform and bright white‐light‐emission under UV irradiation was obtained (Figure 5B). The construction of a remote planar white‐light‐emitting device can be achieved by coating the prepared GQD‐MF‐PDMS film on a UV LED chip (Figure 5C). Using commercially available UV‐LED chips (LED ENGIN, LZ1‐00UV00, 365 nm UV LED with 1200 mW flux output at 2.7W power dissipation) and using 5 × 5 × 0.3 mm GQD‐MF‐PDMS films covered on the top of the UV LED, white LED devices were obtained and tested. Under working current of 700 mA, average luminous efficacy of the prepared white LEDs is 31 ± 4 lm W^−1^, which is comparable to that of some commercial white LED products. This demonstrated the potential of GQD‐MF microspheres for solid‐state luminescent materials and LED devices.

In conclusion, the controlled aggregation of fluorescent nanomaterials by polymer–nanomaterial interaction to regulate their luminescent properties has been demonstrated. GQD‐MF microspheres with a high‐quality white emission were prepared through polymer‐mediated GQD aggregation and encapsulation. Using PVA as a dispersant, GQDs with up to 55% QY and a good dispersibility were synthesized from the hydrothermal reaction between citric acid and ethylenediamine. The obtained GQDs were then mixed with a water‐soluble MF prepolymer. In solution, the negatively charged GQDs were adsorbed and aggregated with the branched cationic MF prepolymer. The extension of the emission spectrum was caused by aggregation. Under acidic catalysis and heating, the GQD‐MF prepolymer complex was further polymerized and cross‐linked to form encapsulated GQD‐MF microspheres. By adjusting the amount of GQDs added, the aggregation state of the GQDs in the MF microspheres, and consequently their emission color, was effectively regulated. Using GQD doping concentrations between 2 wt% and 4 wt%, GQD‐MF microspheres with QY values from 0.83 to 0.43, CIE coordinates from (0.28, 0.28) to (0.33, 0.32), and CRI values from 0.75 to 0.88 were obtained. The prepared GQD‐MF microspheres had a high‐quality white‐light emission and excellent stability. They could also be dispersed in various solvents and polymers, demonstrating their significant potential for the fabrication of luminescent materials and devices.

## Experimental Section

*Materials*: Melamine, paraformaldehyde, citric acid monohydrate, ethylenediamine, PVA 203, fluorescein, and Rhodamine 110 were purchased from Aladdin Chemistry Co., Ltd. THF, DMF, DMSO, dichloromethane, chloroform, and ethanol were purchased from Sinopharm Chemical Reagent Co., Ltd. PMMA (average molecular weight = 350 000 kDa) was purchased from J&K Chemical Ltd. The SYLGARD 184 silicone elastomer kit was purchased from Dow Corning. All chemicals used were of analytical grade and were used without further purification. Deionized water was used in all experiments.

*Preparation of the GQDs*: A total of 1.26 g of citric acid monohydrate, 1.08 g of ethylenediamine, and 0.15 g of PVA 203 were dissolved in 30 mL of water, sealed, and heated in a 50 mL Teflon‐lined stainless autoclave to 160 °C for 4 h. The prepared GQDs were centrifuged and washed with ethanol three times before being dried in air at 60 °C for 8 h. The GQDs were then dispersed in water to prepare a 0.1 wt% GQD solution.

*Preparation of the MF Prepolymer Solution*: A total of 2.6 g of melamine was mixed with 3.7 g of paraformaldehyde and 100 mL of water. The mixture obtained was heated to 50 °C with magnetic stirring for 40 min. The transparent MF prepolymer solution obtained was filtered and stored at 4 °C.

*Preparation of the GQD‐MF Microspheres*: GQD‐MF microspheres with a GQD doping concentration of 2.0 wt% were prepared as follows. A total of 15 mL of GQD solution (0.1 wt%) and 15 mL of MF prepolymer solution (60 mg mL^−1^) were mixed using magnetic stirring for 10 min at room temperature. Hydrochloric acid (1.0 mole L^−1^) was added drop by drop to adjust the pH of the solution to 3.2. The mixed solution obtained was then heated to 95 °C in a water bath under magnetic stirring for 40 min to form the GQD‐MF microspheres. The GQD‐MF microspheres prepared were then centrifuged and washed with ethanol three times before being dried in air at 120 °C for 8 h. The GQD‐MF microspheres with higher GQD doping concentrations were prepared with GQD solutions of higher concentrations.

*Preparation of the GQD‐MF‐PDMS Film*: A total of 0.05 g of GQD‐MF microspheres and 1.0 g of SYLGARD 184 silicone elastomer mixture were dispersed in 5 mL of dichloromethane to prepare the GQD‐MF‐PDMS film. The microsphere suspension obtained was dispersed by ultrasonication, poured into a flat glass dish, and dried with air at 80 °C for 4 h.

*Characterization*: Zeta potential of the GQDs and MF prepolymer in solutions of varied pH (pH 2 to pH 12) were measured with a Malvern Zetasizer Nano ZS90 DLS system. The UV–vis absorption of the GQD solution, mixture of GQDs and MF prepolymer, and series of GQD‐MF microsphere samples were analyzed with a PE Lambda950 UV–VIS–NIR Spectrometer. The HRTEM images were obtained using a JEOL JEM‐2100F field‐emission electron microscope with a 200 kV electron source. The average sizes of the samples were measured by counting 200 individual particles in the TEM images. SEM images were obtained using a Hitachi S‐2700 scanning electron microscope with a 200 kV electron source. The average sizes of the samples were measured by counting 200 individual particles in the SEM images. AFM images were captured on a Bruker Multimode 8 in tapping mode. X‐ray photoelectron spectra were obtained using a Thermo Scientific ESCALAB 250 Multitechnique Surface Analysis with an a1 K‐alpha X‐ray monochromator with a pass energy of 20 eV. FTIR spectra were obtained with a Bruker Optics Tensor 27 spectrometer. Fluorescence spectra, QY, and lifetime measurements were performed using HORIBA Jobin Yvon FluoroMax‐4 and Edinburgh FLS 980 fluorescence spectrophotometers with calibrated integrating sphere. Fluorescence microscopy images were captured with a Leica DMi8 inverted microscope. Laser scanning confocal microscopy images were captured with a Leica TCS SP8 STED 3X super‐resolution confocal microscope.

## Conflict of Interest

The authors declare no conflict of interest.

## Supporting information

Supplementary
