# Supplementary material for: White‐Light‐Emitting Melamine‐Formaldehyde Microspheres through Polymer‐Mediated Aggregation and Encapsulation of Graphene Quantum Dots
Source: Adv Sci (Weinh). 2018 Nov 22;6(2):1801432. doi: 10.1002/advs.201801432 (PMC6343069; doi:10.1002/advs.201801432)
Supplement: Supplementary file 1 — Supplementary [file ADVS-6-1801432-s001.pdf]

Copyright WILEY-VCH Verlag GmbH & Co. KGaA, 69469 Weinheim, Germany, 2018.

ADVANCED SCIENCE
Open Access

Supporting Information

for *Adv. Sci.,* DOI: 10.1002/advs.201801432

White-Light-Emitting Melamine-Formaldehyde Microspheres through Polymer-Mediated Aggregation and Encapsulation of Graphene Quantum Dots

*Youshen Wu, Hui Zhang, Aizhao Pan, Qi Wang, Yanfeng Zhang, Guijiang Zhou, and Ling He**

Copyright WILEY-VCH Verlag GmbH & Co. KGaA, 69469 Weinheim, Germany, 2016.

Supporting Information

White-light-emitting melamine-formaldehyde microspheres through polymer mediated aggregation and encapsulation of graphene quantum dots

Youshen Wu, Hui Zhang, Aizhao Pan, Qi Wang, Yanfeng Zhang, Guijiang Zhou and Ling He*

Dr. Y. Wu, Dr. A. Pan, Q. Wang, Dr. Y. Zhang, Prof. G. Zhou, Prof. L. He
Department of Chemistry, School of Science, Xi'an Jiaotong University, Xi'an 710049, P. R. China
E-mail: Heling@mail.xjtu.edu.cn

Dr. H. Zhang
Key Laboratory of Biomedical Information Engineering of Education Ministry, Xi'an Jiaotong University, Xi'an 710049, P. R. China

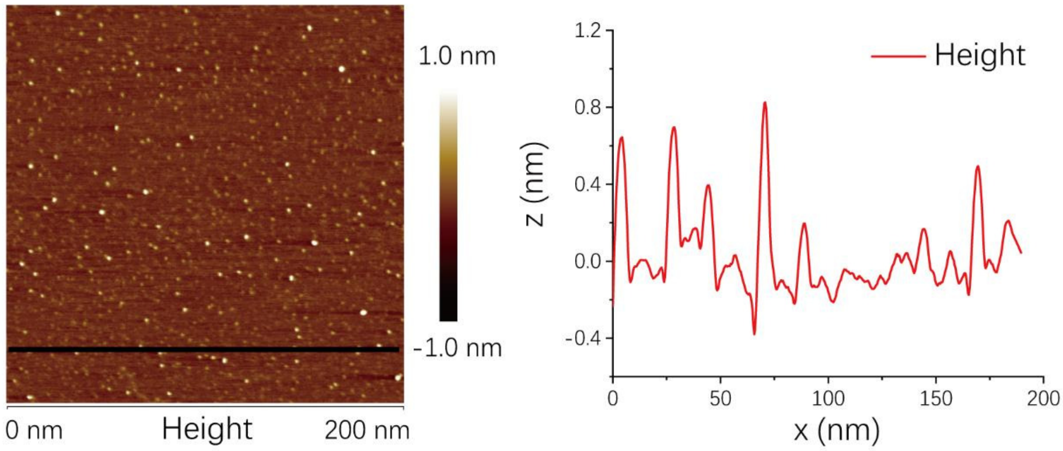

Figure S1. AFM image and topographic heights of the GQDs.

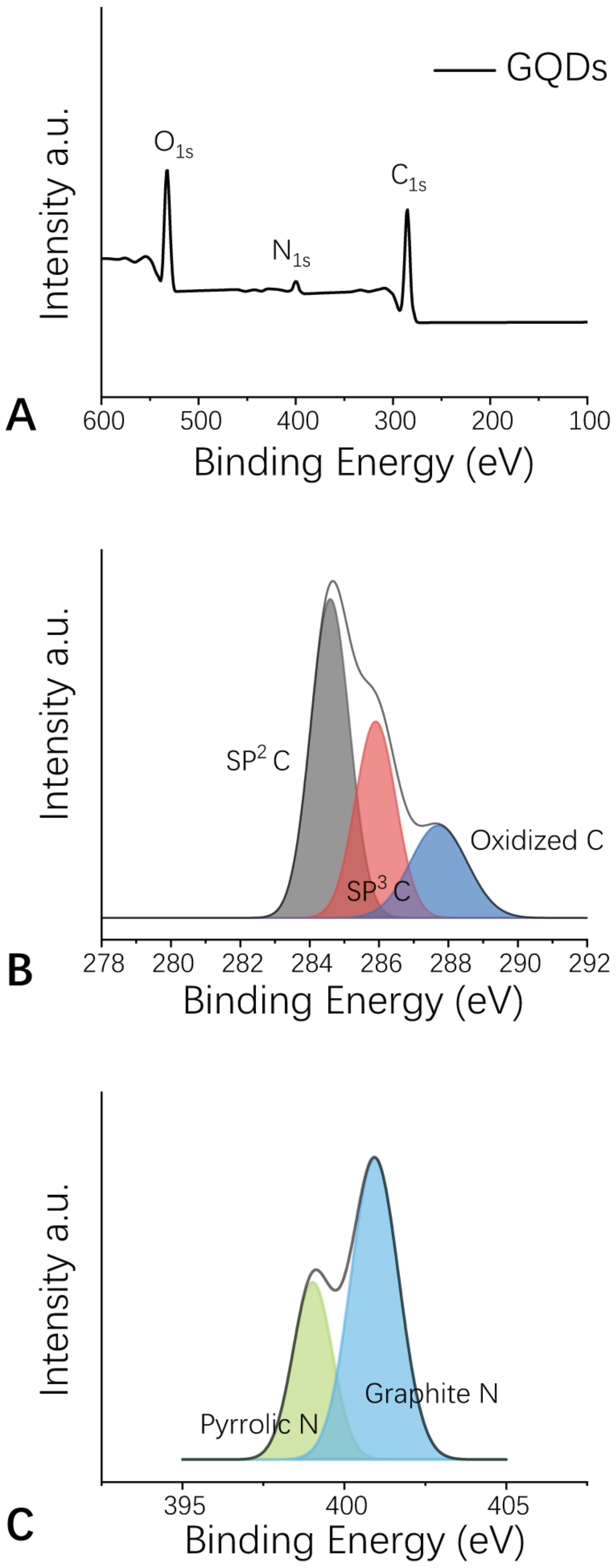

Figure S2. XPS spectra (full survey, C1s, N1s, O1s) of the GQDs (A). Deconvoluted XPS spectra for C1s (B) and N1s (C).

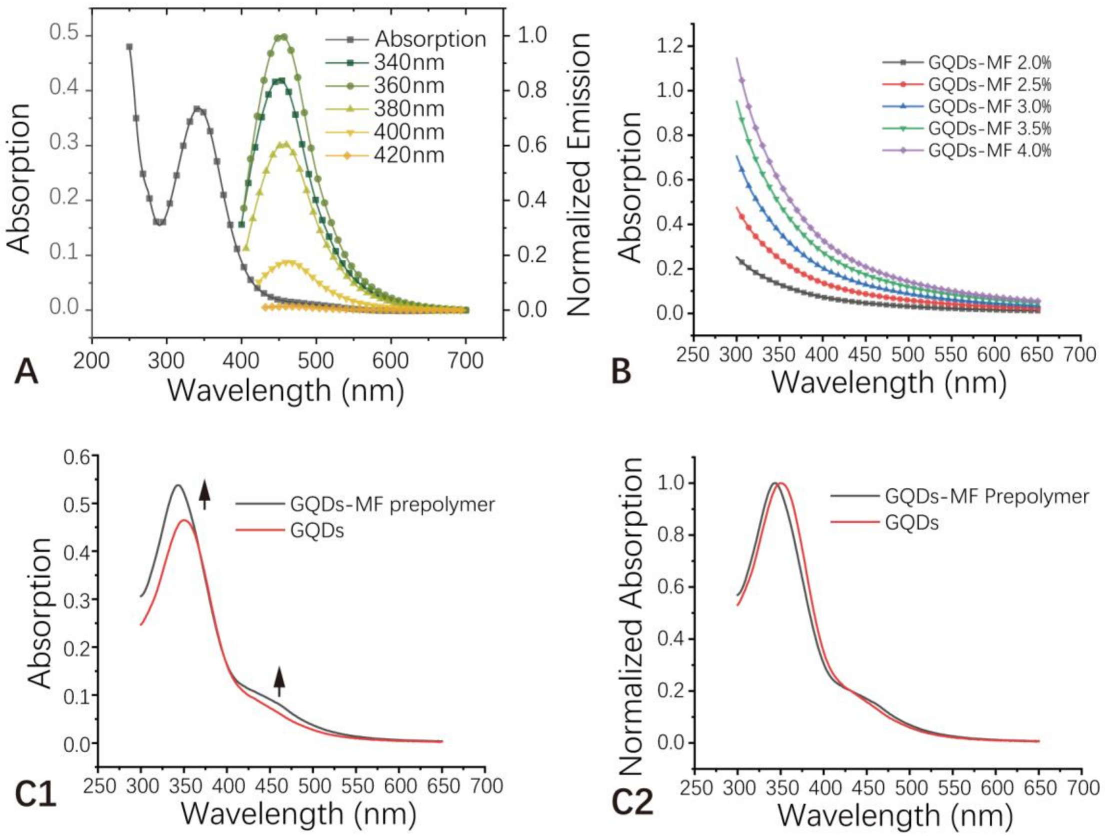

Figure S3. UV-Vis absorption and fluorescence spectra of the GQDs solution, excited at different wavelengths (A). UV-Vis absorptions of the series of GQDs-microspheres (B), the GQDs solution (0.1% wt.) and of the GQDs-MF prepolymer mixture (C1 and C2).

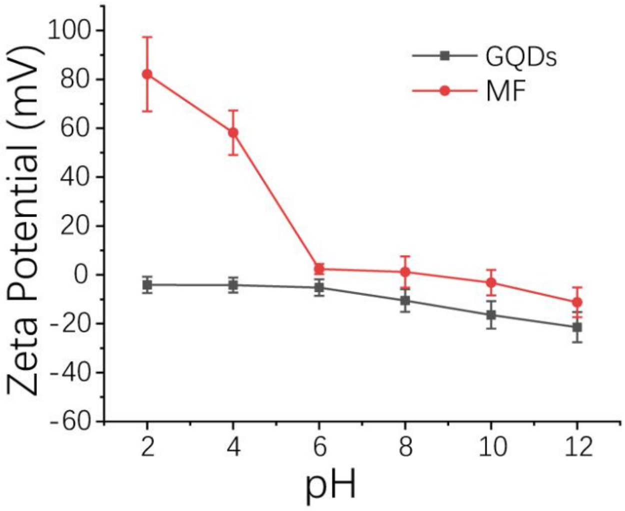

Figure S4. pH-zeta potential curves of GQDs and MF prepolymer in aqueous solutions

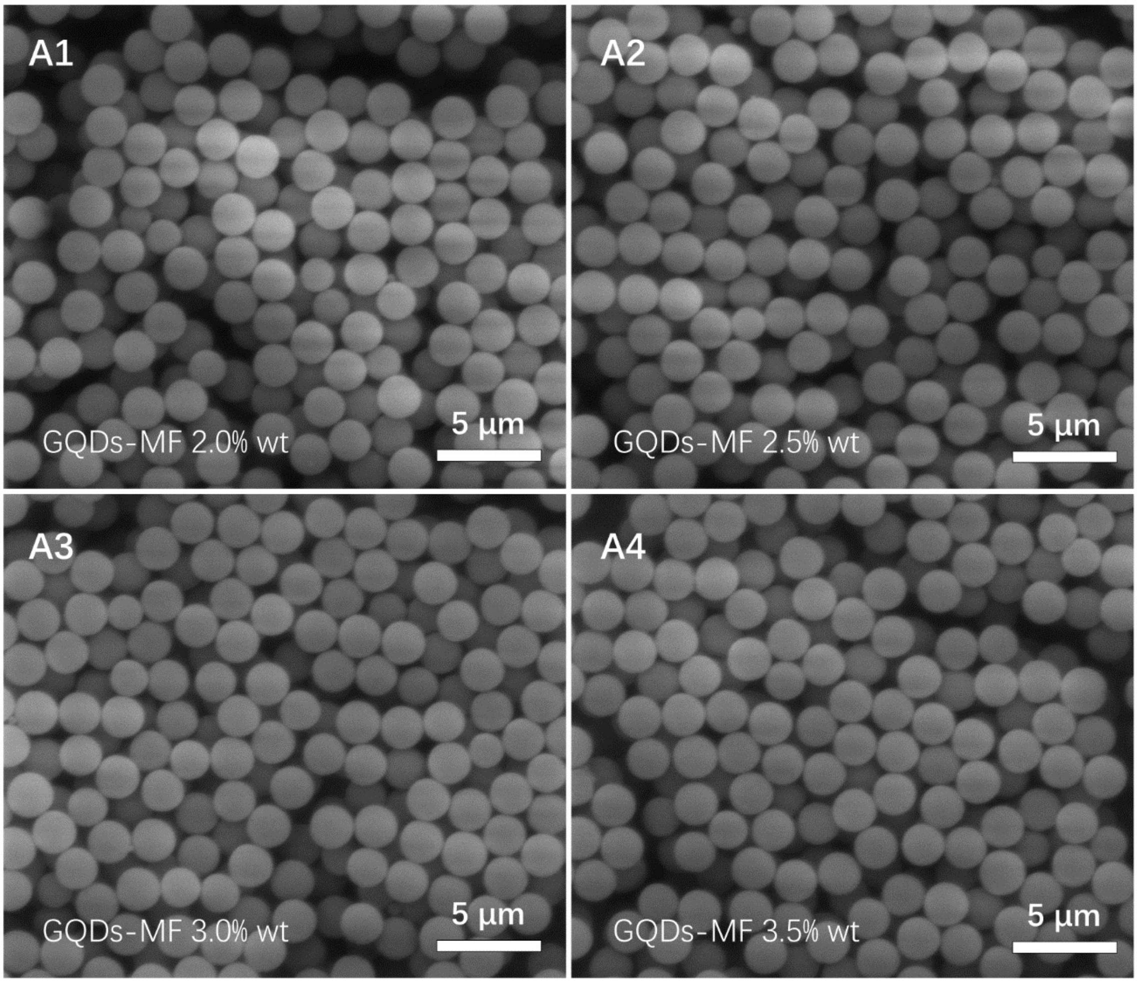

Figure S5. SEM images of GQDs-MF microsphere samples prepared with varied GQDs doping concentrations.

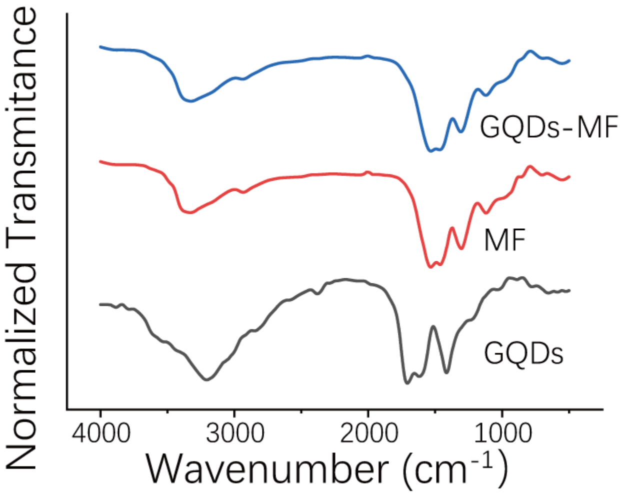

Figure S6. FTIR spectra of the GQDs, GQDs-MF microspheres and blank MF microspheres.

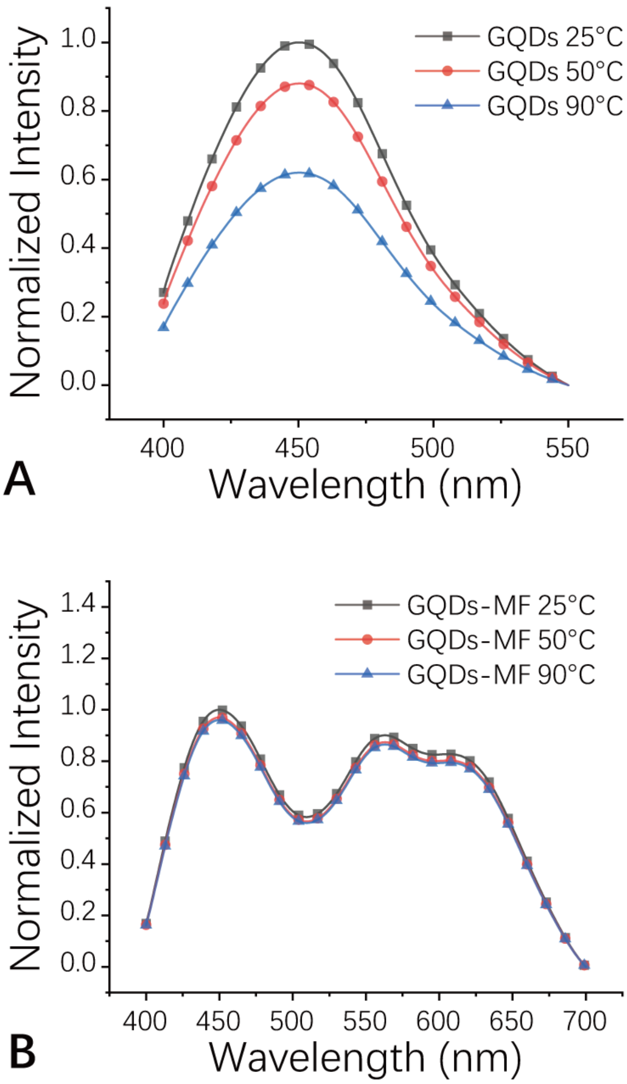

Figure S7. Emissions spectra of the GQDs solution (A) and aqueous suspension of GQDs-MF microspheres at varied temperatures, excited by 360 nm.

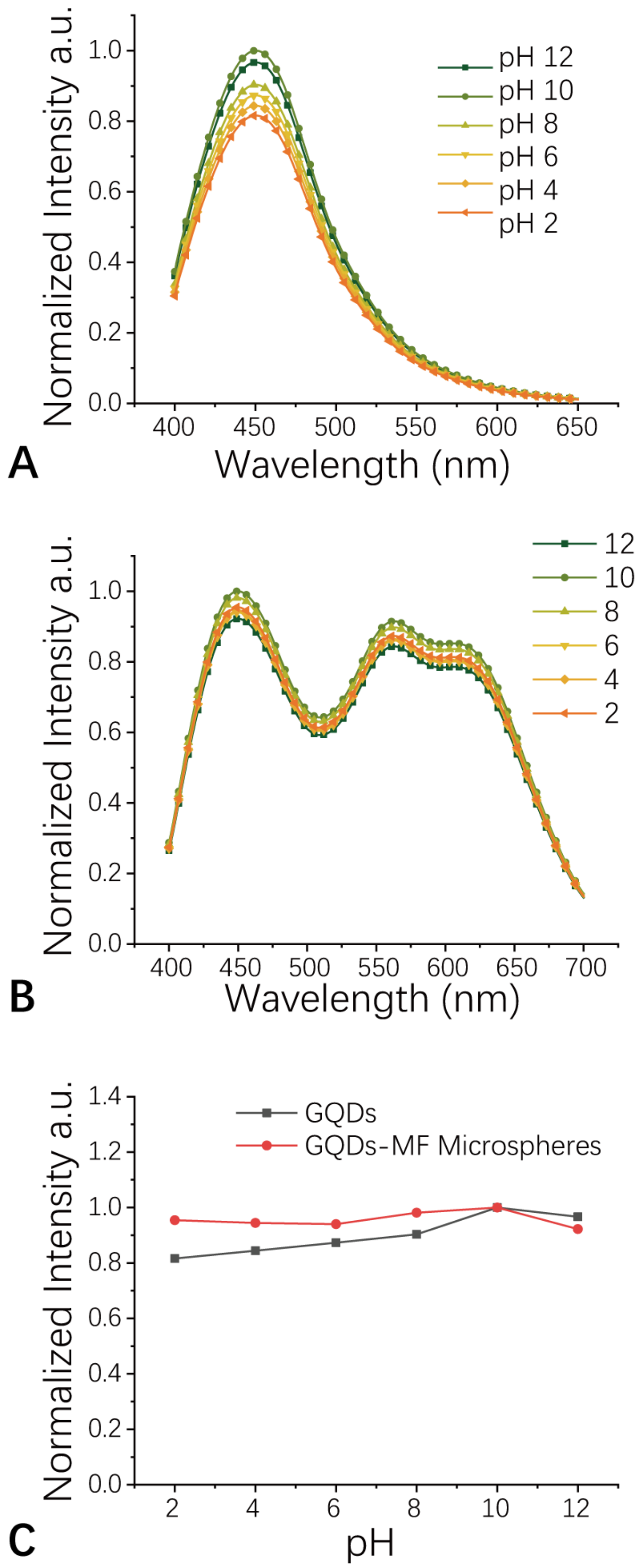

Figure S8. Emission spectra and maximum emission intensity of the GQDs solution and aqueous suspension of GQDs-MF microspheres in solutions of different pH, excited at 360 nm.

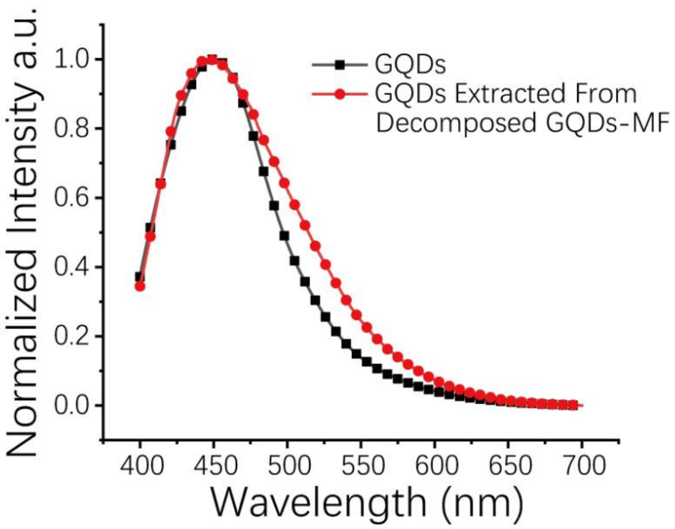

Figure S9. Emission spectra of the GQDs and GQDs extracted from the decomposed GQDs-MF microspheres, the spectra are normalized by maximum intensity, excited at 360 nm.

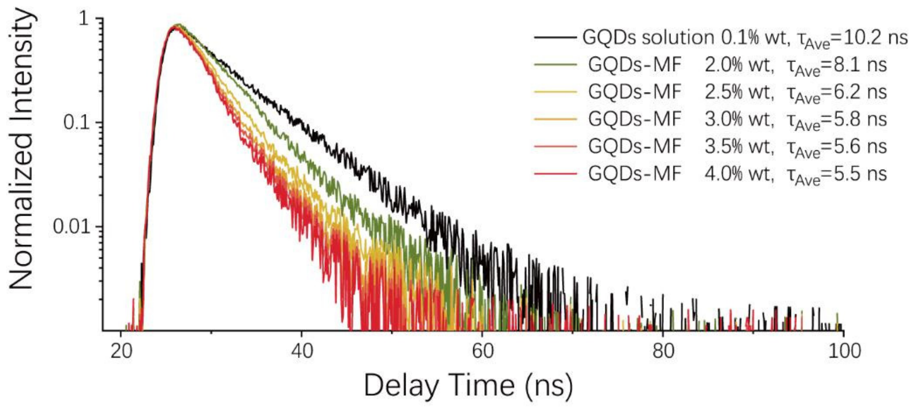

Figure S10. Time-resolved luminescence decay curves of the GQDs solution and series of GQDs-MF microspheres. The emissions were collected at 450 nm, excited at 340 nm.

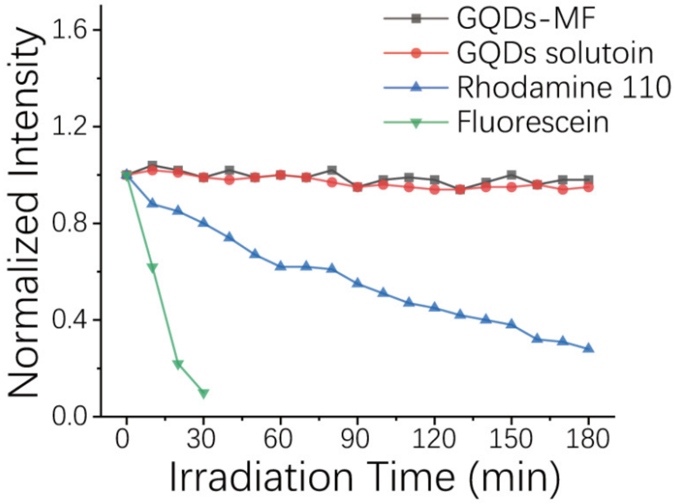

Figure S11. A comparison of the photo-stability of the GQDs solution, GQDs-MF microspheres, Rhodamine 110, and Fluorescein, under UV irradiation by a 300W high pressure mercury lamp

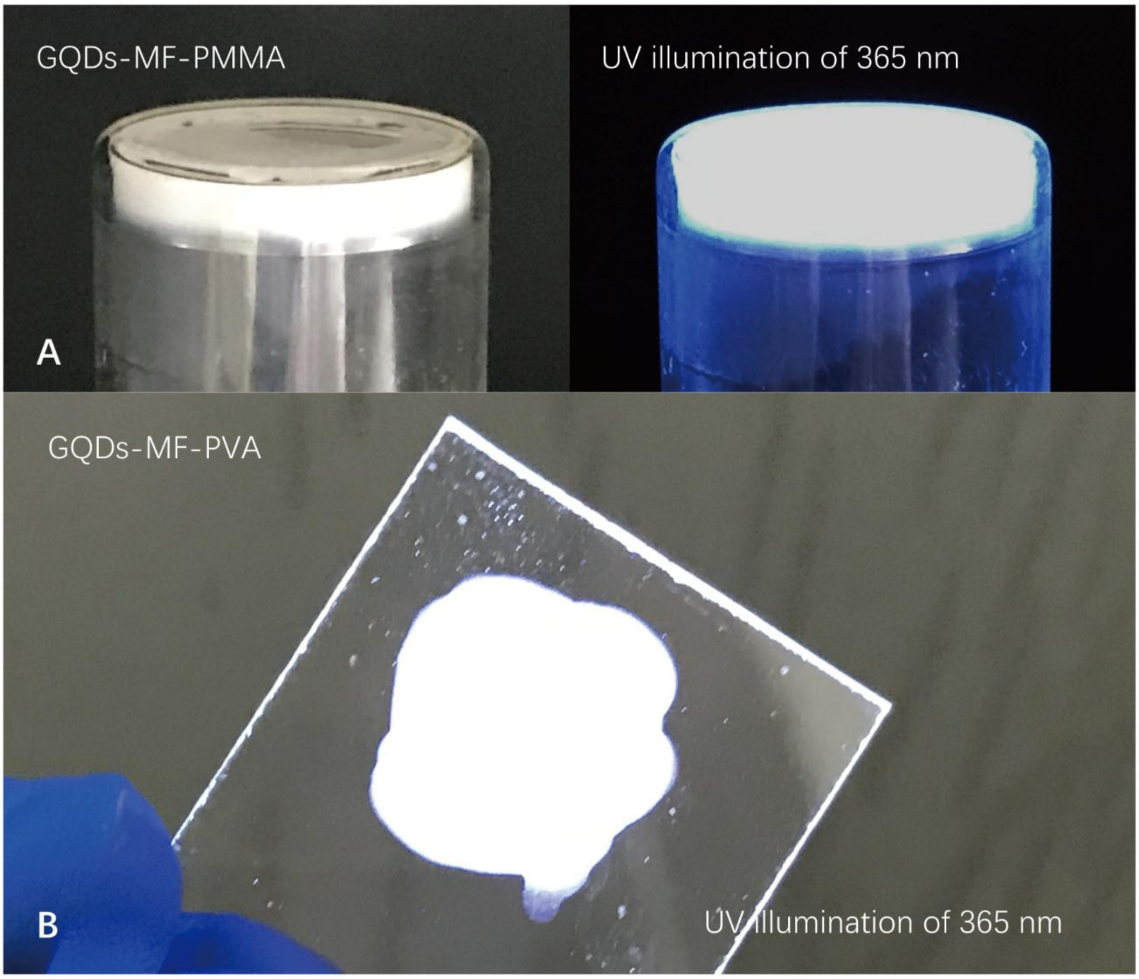

Figure S12. White-light-emitting block and film prepared with GQDs-MF-PMMA and GQDs-MF-PVA materials.

Table S1. QY, CCT, CRI and CIE coordinates for the GQDs solution and GQDs-MF microspheres prepared with different GQDs doping concentrations.

Doping Concentration	QY	CCT (K)	CRI	CIE
0.1% wt solution	0.55	NA	NA	(0.16, 0.14)
2.0% wt.	0.83	10810	0.75	(0.28, 0.28)
2.5% wt.	0.74	7814	0.81	(0.30, 0.29)
3.0% wt.	0.62	6653	0.84	(0.31, 0.31)
3.5% wt.	0.51	5969	0.87	(0.32, 0.32)
4.0% wt.	0.43	5638	0.88	(0.33, 0.32)

Table S2. A comparison of some recently developed GQDs based white-light-emitting materials.

Author and Date	CIE	CRI	QY	EQE	Ref.
**This work**	(0.28, 0.28); (0.30, 0.29); (0.31, 0.31); (0.32, 0.32); (0.33, 0.32);	0.75-0.88	43-83%	NA	This work
**P. Dong et. al. 2017**	(0.33, 0.36)	83.9	3.62%	NA	23
**Z. Luo et. al. 2016**	(0.24, 0.25); (0.25, 0.27); (0.26, 0.28); (0.27, 0.29)	NA	NA	0.24 - 0.19%	46
**T. Ghosh et. al. 2015**	(0.29, 0.34)	NA	1-2%	NA	25
**R. Sekiya et. al. 2014**	(0.24, 0.27); (0.34, 0.40); (0.34, 0.39); (0.32, 0.38)	NA	1-2%	NA	21
**C.M. Luk et. al. 2012**	(0.33. 0.38)	72.0	12%	NA	24
